# Differential moderation effects of ApoE and 5-HTTLPR genotypes on social vulnerability in predicting mortality among community-dwelling middle-aged and older adults: a nationwide population-based study

**DOI:** 10.18632/aging.203629

**Published:** 2021-10-14

**Authors:** Hsin-Yu Liu, Li-Ning Peng, Wei-Ju Lee, Ming-Yueh Chou, Chih-Kuang Liang, Fei-Yuan Hsiao, Ming-Hsien Lin, Liang-Kung Chen

**Affiliations:** 1 Aging and Health Research Center, National Yang Ming Chiao Tung University Yangming Campus, Taipei City, Taiwan; 2 Institute of Public Health, National Yang Ming Chiao Tung University Yangming Campus, Taipei City, Taiwan; 3 Center for Geriatrics and Gerontology, Taipei Veterans General Hospital, Taipei City, Taiwan; 4 Center for Geriatrics and Gerontology, Kaohsiung Veterans General Hospital, Kaohsiung City, Taiwan; 5 Department of Family Medicine, Taipei Veterans General Hospital Yuanshan Branch, Yilan City, Taiwan; 6 Graduate Institute of Clinical Pharmacy, College of Medicine, National Taiwan University, Taipei City, Taiwan; 7 School of Pharmacy, College of Medicine, National Taiwan University, Taipei City, Taiwan; 8 Department of Pharmacy, National Taiwan University Hospital, Taipei City, Taiwan; 9 Taipei Municipal Gan-Dau Hospital (Managed by Taipei Veterans General Hospital), Taipei City, Taiwan; 10 Department of Family Medicine, Kaohsiung Veterans General Hospital Pingtung Branch, Pingtung County, Taiwan

**Keywords:** social vulnerability, survival, gene-environment interaction, serotonin transporter polymorphism (5-HTTLPR), apolipoprotein E gene (ApoE)

## Abstract

Aging is a dynamic complex process involving social vulnerability over time. The social vulnerability index (SVI) was developed that predicted adverse health outcomes. This study examined effects between SVI status and two genotypes, apolipoprotein E (ApoE) and Serotonin transporter genotyping (5-HTTLPR), on all-cause mortality. Data from the Social Environment and Biomarkers of Aging Study (SEBAS) were obtained, and SVI was constructed using 32 self-reported items of social determinants. Data from 985 participants (age: 65.73 ± 9.47 years, 54.62% males) were obtained for analysis, and the median SVI was 0.35 (IQR 0.29–0.42) with a near normal distribution. Participants with a higher SVI were more likely to be women and have poor cognitive function, more depressive symptoms and poor physical function. Adjusted for age and sex, each incremental deficit in SVI was associated with a 12% increase in mortality risk (HR: 1.12, 95% CI: 1.04–1.20, *p* = 0.002). An interaction was found between ApoE and SVI but not 5-HTTLPR. The strata-specific hazard ratio confirmed that associations between SVI and mortality was only in non-ε4 carriers (HR: 1.15, 95% CI: 1.07–1.24, *p* < 0.001), and SVI did not significantly predict mortality among ε4 carriers (HR: 0.84, 95% CI: 0.65–1.10). Differential SVI effects on mortality among middle-age and older adults were identified. In conclusion, a higher SVI was associated with all-cause mortality among middle-aged and older adults, and the association was moderated by ApoE genotypes but not 5-HTTLPR. Further study is needed to evaluate the clinical efficacy of healthy aging intervention programs considering gene-environment interactions and social vulnerability.

## INTRODUCTION

The World Health Organization published the World Report on Aging and Health that proposed the conceptual framework of healthy aging to promote late life well-being through functional ability and intrinsic capacity [1]. One of the key issues of promoting healthy aging involves the development and maintenance of functional ability and prevention of declines in late life. Previous studies have shown that disability significantly outweighs multimorbidity in quality of life and the risk of mortality [2, 3]. However, age-related functional declines are progressive and are affected by a great variety of determinants. Along with aging, reductions in physiological reserve, the development of chronic conditions, socioeconomic status and other factors are often intertwined to cause functional declines and disability [4, 5]. In particular, social inequality, social engagement, social cohesion, sense of life control, social networks, and socioeconomic status all play crucial roles in the health of older people [6]. Compared to health domains, studies have shown that social factors were more influential on health outcomes and mortality, and inhabitants of socially deprived areas had a higher mortality rate than communities with higher incomes under the same universal health coverage [7–9].

The social vulnerability index (SVI) is an aggregation of several items that reflects different aspects of social factors interacting with health and has been validated in predicting cognitive declines and mortality among community-dwelling older adults [10, 11]. The SVI was developed based on the cumulative deficit theory that focused on deficits of social domains without biomedical dimensions. SVI values tend to be distributed normally, which differs from the strong right skewness of the frailty index (FI), which comprises a wide dimension of health determinants based on the same cumulative deficit theory [12]. The differences in the distribution between FI and SVI suggested the unique impacts of social vulnerability on the health of older individuals. Nevertheless, social factors may interact with genetic backgrounds to cause different health outcomes, especially in mental health [13]. Gene-environment (GxE) interactions have been observed due to the success of the Human Genome Project [14]. For example, the ε4 allele of apolipoprotein E (ApoE) has been reported to increase the risk of Alzheimer’s disease, cognitive decline, and coronary artery disease [15–17]. The mortality risk increased in older people with ε4 allele whereas ε2 allele was associated with decreased mortality [18]. Moreover, physical frailty accelerates cognitive decline among ApoE ε4 allele carriers [19], but cognitive and physical activities slow the onset of dementia and reduce brain pathology [20, 21]. Serotonin transporter genotyping (5-HTTLPR) also revealed similar conditions. Short (S) allele carriers were more susceptible to adverse family environments and stressful life events and exhibited an increased risk for depressed symptoms than long (L) allele carriers [22, 23]. Goldman et al. further revealed depressed mood was more severe in those with S/S and S/L genotypes experiencing major trauma events than those with S/XL, L/L, and L/XL genotypes [24]. Besides, the developments of cognitive impairment and depressive symptoms are the results of GxE interactions, especially social factors, but studies examining the interactions between social vulnerability and ApoE and 5-HTTLPR genotypes are scarce. Hence, this study aimed to explore the clinical outcomes of the GxE interactions between social vulnerability and ApoE and 5-HTTLPR genotyping among community-dwelling middle-aged and older adults using a nationwide population-based cohort study. Our hypothesis is that social vulnerability may predict mortality and the effect is moderated by different genotype of ApoE or 5-HTTLPR.

## METHODS

### Study population and study design

The data were retrieved from the second wave of the Social Environment and Biomarkers of Aging Study (SEBAS) conducted in 2006. As an extension of the Taiwan Longitudinal Study of Aging (TLSA: also called the Survey of Health and Living Status of the Near Elderly and Elderly), SEBAS is a longitudinal survey with national representativeness that aims to determine the interrelationships between the social environment and biomarkers in the aging process. Details about sample selection, participation, and attrition for SEBAS and TLSA have been described previously [25, 26]. A total of 1284 participants from subsamples of the SEBAS 2000 and 2003 TLSA surveys underwent complete exams, and face-to-face interview data comprised the SEBAS 2006 dataset.

The STROBE guidelines were applied for the observational design and reporting format of this study [27]. All participants signed informed consent forms, and the study protocol was approved by The Joint Institutional Review Board of Taiwan (06-044-C).

### Social vulnerability index (SVI) construction

The SVI was developed based on cumulative deficit theory [12]. Among all study variables of SEBAS, we selected 32 parameters from social perspectives, including social support, social engagement, personal mastery, marital status, education attainment, socioeconomic status and two instrumental activities of daily living items associated with community participation, to construct the SVI (Supplementary Table 1). For binary social deficits in each respondent, a score of 0 was assigned if the deficit was absent, and 1 was assigned if the deficit was present. Intermediate values were assigned scores ranging from 0 to 1 to generate an ordered response. For example, for the “living alone” question, an individual scored 1 if the answer was “yes” and 0 if the answer was “no”. As another example, the question “in the past week, do you feel people around you weren’t nice to you” had four response categories. A score of 0 was assigned if the answer was “no”, 0.33 for “rarely”, 0.66 for “sometimes” and 1 for “often”. Subjects with >20% missing values of the deficit items were excluded from the analysis. The SVI value was obtained by the sum of the deficit scores and then divided by the total number of total deficit items to yield a comparable score ranging from 0 to 1. The higher the SVI score, the more severe the increase in social vulnerability.

### Genotype classification

Two genotypes of the serotonin transporter polymorphism (5-HTTLPR) and apolipoprotein E gene (ApoE) were explored in this study by extracting DNA from venous blood samples and then amplifying it by polymerase chain reaction. Three allele variants of 5-HTTLPR were identified: short, long, and extralong. Thus, the five following genotypes were classified: S/S, S/L, L/L, S/XL, and L/XL (no respondents were XL alleles). Using the same study sample, Goldman et al. found depressed mood was associated with S/S and S/L genotypes after experiencing major trauma events [24]. Therefore, we combined the S/S and S/L genotypes together as a high-risk group with a score of 1, and the other groups were assigned a score of 0. On the other hand, three alleles of the ApoE gene, ε2, ε3 and ε4, yielded six genotypes. As both positive and negative effect of ε2/ε4 genotype on mortality has been reported [18], we further exclude those with ε2/ε4 genotype and classified other genotypes as follows. Individuals with ε3/ε4 and ε4/ε4 were classified into high risk group with a score of 1, and the other individuals without ε4 allele were classified into another group with a score of 0.

### Measurements for other covariates

Demographic characteristics of all subjects, e.g., age, sex, and years of education, were included in the analysis. Smoking and drinking status were based on tobacco and alcohol consumption in the previous six months. Depressive symptoms and cognitive function were examined using a 10-item Center for Epidemiologic Studies Depression (CES-D) scale and 8-item Chinese-version Short Portable Mental Status Questionnaire (SPMSQ) [28, 29]. Multimorbidity was defined as having two or more chronic diseases [30]. Physical function was evaluated by applying the Katz Index of Independence in Activities of Daily Living (ADLs) and the Lawton Instrumental Activities of Daily Living (IADLs).

### Statistical analysis

In this study, descriptive statistics were expressed as the means ± standard deviation for continuous variables and proportions for categorical variables. Student’s ANOVA test and chi-square test were applied to compare continuous and categorical variables between tertiles of SVI. Kaplan-Meier curves were applied for survival analysis to determine the trends of different SVI status and genotypes. Cox proportional hazards regression was performed to further assess the association between the SVI and overall mortality with strata-specific hazard ratios of different genotypes. All the models were adjusted for age and sex (model 1); age, sex, educational level, multimorbidity, SPMSQ, and ADL (model 2); and age, sex, educational level, multimorbidity, SPMSQ, ADL, 5-HTTLPR and APOE (model 3). CES-D and IADL were not included in the statistical model as several questions related to CES-D and IADL has been used in the development of SVI. Adjusting CES-D and IADL to the model may result in over-adjustment. We also conducted a subgroup analysis on the older adults (≥65 years) and middle-aged adults (50–64 years) to see whether these associations varied by age.

All statistical analyses were performed using STATA version 15 (StataCorp LLC. College Station, Texas). For all tests, a two-tailed *p*-value < 0.05 was considered statistically significant.

## RESULTS

**Figure 1:**
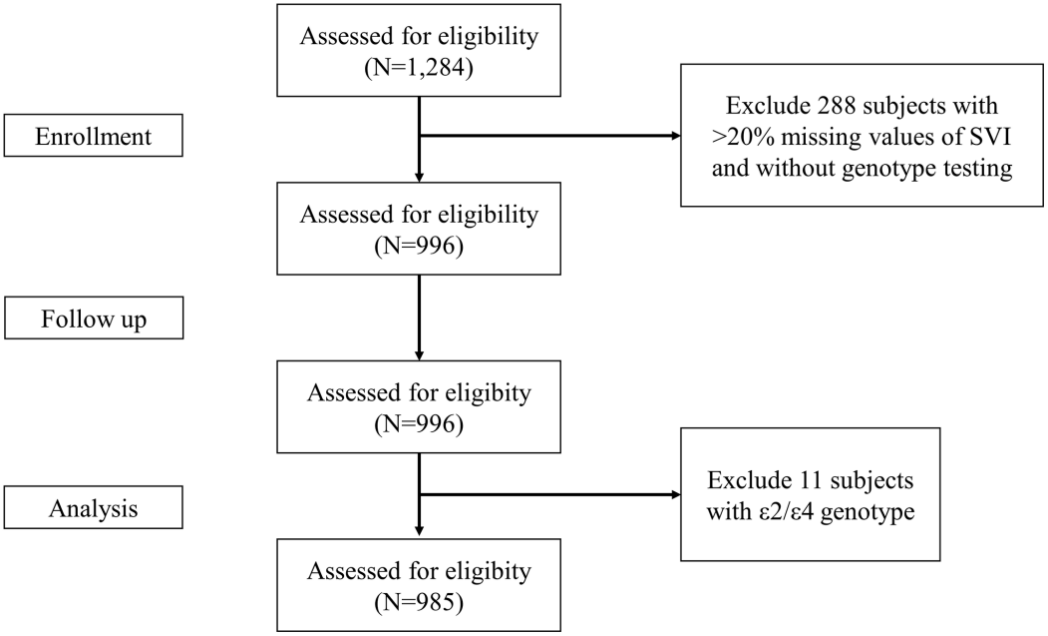
The flow chart for inclusion of study participants.

**Figure 2:**
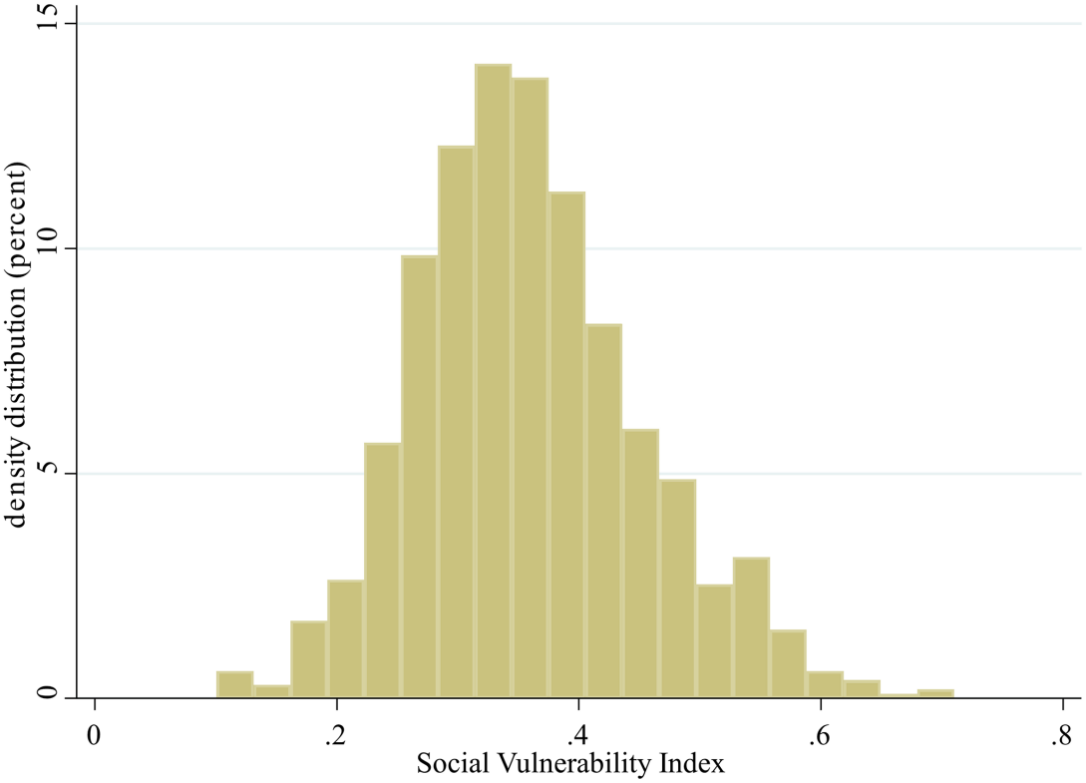
Distribution of the social vulnerability index.

Among 1,284 participants recruited in the SEBAS 2006 dataset, data from 985 participants were obtained for data analysis after excluding 299 subjects with >20% missing values, those without genotype testing and those with ε2/ε4 genotype (Figure 1). The mean age of all participants was 65.73 ± 9.47 years (from 53 to 85 years old) with 538 males (54.62%). The median constructed SVI was 0.35 (IQR 0.29–0.42) with a near normal distribution (Figure 2). In Table 1, we stratified participants by SVI tertile, and significant differences in age, sex, educational level, CES-D, SPMSQ, multimorbidity, ADL, and IADL were revealed across different levels of SVI. We found that the SVI increased with age and that women had a higher SVI than men. In addition, participants with a lower SVI had better cognitive function, higher educational level, and lower CES-D scores. Participants with higher SVI had poor functional status in ADLs and IADLs. In terms of variations in genotyping between tertiles of SVI, only ApoE showed statistical significance, not 5-HTTLPR.

**Table 1:** Comparisons of characteristics of participants of the social vulnerability index (SVI) tertiles.

		**Total** **(*N* = 985)**		**Tertile of SVI**						***p*-value**
				**Low** **SVI ≦ 0.31** **(*N* = 330)**		**Intermediate** **0.31 < SVI ≦ 0.39** **(*N* = 327)**		**High** **0.39 < SVI** **(*N* = 328)**		
Age (years)		65.73	±9.47	63.15	±8.63	65.07	±9.08	69.00	±9.75	<0.001
Sex (Men)		538	(54.62)	196	(59.39)	190	(58.1)	152	(46.34)	0.001
Education level (>6 years)		375	(38.07)	179	(54.24)	118	(36.09)	78	(23.78)	<0.001
CES-D^a^		4.69	±5.50	1.66	±2.43	3.32	±3.50	9.25	±6.46	<0.001
SPMSQ^b^		0.54	±1.10	0.23	±0.52	0.42	±0.83	0.98	±1.55	<0.001
Multimorbidity (≥2 chronic conditions)		655	(66.63)	194	(58.79)	215	(65.95)	246	(75.23)	<0.001
ADL^c^		5.78	±0.96	5.98	±0.15	5.96	±0.28	5.40	±1.57	<0.001
IADL^d^		5.32	±1.37	5.85	±0.42	5.62	±0.87	4.49	±1.90	<0.001
5-HTTLPR gene	S/XL, L/L,L/XL	164	(16.65)	62	(18.79)	52	(15.9)	50	(15.24)	0.43
	S/S, S/L	821	(83.35)	268	(81.21)	275	(84.1)	278	(84.76)	
APOE gene	ε2ε2, ε3ε2, ε3ε3	852	(86.5)	294	(89.09)	287	(87.77)	271	(82.62)	0.037
	ε4ε3, ε4ε4	133	(13.5)	36	(10.91)	40	(12.23)	57	(17.38)	

^a^Center for Epidemiologic Studies Depression (CES-D) scale. ^b^Short Portable Mental Status Questionnaire (SPMSQ). ^c^Activity of daily living. ^d^Instrumental activity of daily living.

**Figure 3:**
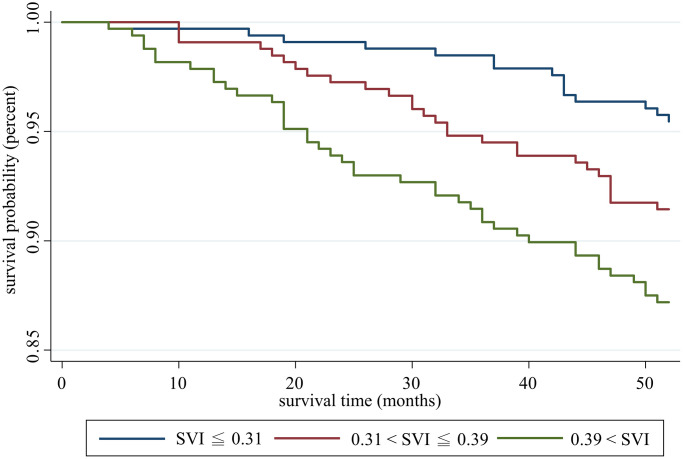
Survival analysis of participants with different social vulnerability index statuses.

The average time of follow-up was 50.11 ± 7.40 months, and 85 deaths were recorded. Kaplan-Meier analysis showed a significantly lower survival probability as the SVI level increased (Figure 3). In Cox proportional hazard regression, after adjustment for age and sex, the association was significant. Specifically, each increase in 1 deficit item in SVI increased the mortality risk by 12% (hazard ratio [HR]: 1.12, 95% CI: 1.04–1.20, *p* = 0.002). In model 2 and model 3, we further adjusted education level, multimorbidity, SPMSQ, ADL and genotypes and the SVI remained significantly positively associated with mortality risk, which indicated an independent effect of potential confounders and genotypes (Table 2).

**Table 2:** Cox proportional hazard ratio of social vulnerability index (SVI) and all-cause mortality adjusted by age, sex, educational level, multimorbidity, SPMSQ, ADL and different genotypes (*N* = 985).

**Variables**	**Model 1^a^**		**Model 2^b^**		**Model 3^c^**	
	**HR**	**95% CI**	**HR**	**95% CI**	**HR**	**95% CI**
SVI	1.12	(1.04–1.20)^**^	1.10	(1.01–1.19)^*^	1.10	(1.01–1.19)^*^
Age	1.08	(1.05–1.11)^***^	1.07	(1.04–1.10)^***^	1.07	(1.04–1.10)^***^
Sex	0.54	(0.34–0.86)^*^	0.49	(0.30–0.80)^**^	0.49	(0.30–0.79)^**^
Education level			0.85	(0.51–1.42)	0.85	(0.51–1.41)
Multimorbidity			0.91	(0.54–1.56)	0.93	(0.55–1.59)
SPMSQ			1.20	(1.02–1.40)^*^	1.20	(1.02–1.41)^*^
ADL			1.03	(0.87–1.22)	1.03	(0.86–1.23)
5-HTTLPR					1.09	(0.57–2.06)
APOE					0.63	(0.30–1.31)

^*^p < 0.05; ^**^p < 0.01; ^***^p < 0.001. ^a^Adjusted for age and sex. ^b^Adjusted for age, sex, education level (>6 years), multimorbitidy (≥2 chronic conditions), SPMSQ, ADL. ^c^Adjusted for age, sex, education level (>6 years), multimorbitidy (≥2 chronic conditions), SPMSQ, ADL, APOE and 5-HTTLPR genotypes.

**Figure 4:**
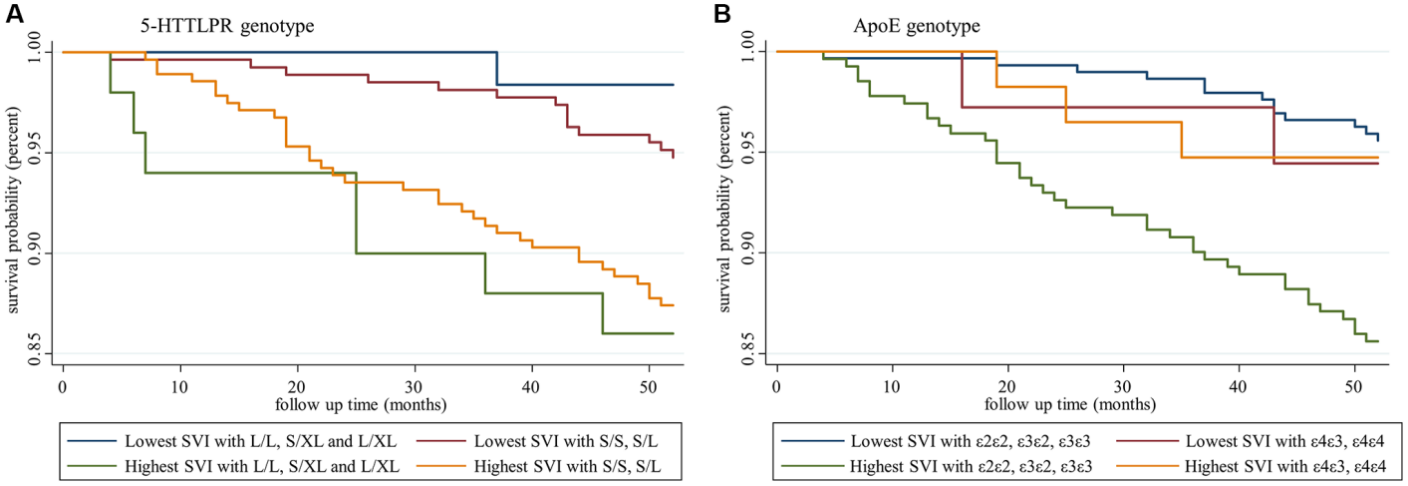
**Survival analysis of participants with different social vulnerability index and genotype statuses.** (**A**) Social vulnerability index and 5-HTTLPR genotypes, and (**B**) social vulnerability index and ApoE genotypes.

To further examine the genotype effects on SVI in predicting mortality, we chose the highest and lowest tertiles of SVI and 5-HTTLPR genotypes from four groups and performed Kaplan-Meier analysis. In Figure 4A, both lowest SVI groups with or without the short allele of 5-HTTLPR exhibited better survival than the highest SVI group, indicating that 5-HTTLPR had no moderation effect on SVI in predicting mortality. We performed a similar analysis for ApoE genotypes and found different results (Figure 4B). Kaplan-Meier analysis revealed significantly lower survival among participants with the highest SVI and without the ε4 allele. Other groups without the ε4 allele but lower SVI showed similar survival trends to those with the ε4 allele at all SVI levels. This effect suggested potential interaction effects of the ApoE genotype on SVI. Therefore, an interaction term was tested, and significant interaction effect was noted between ApoE and SVI but not between 5-HTTLPR and SVI (Table 3). The strata-specific hazard ratio based on the ApoE genotype was calculated, and a significant hazard ratio was found in the non-ε4 allele group, which showed a 15% increased risk of mortality per deficit increase in SVI (Table 4).

**Table 3:** Tests of interaction terms.

**Model**	**LR test^a^**	***p*-Value**
Model^b^ ± social vulnerability index#5-HTTLPR	1.02	0.31
Model^b^ ± social vulnerability index#ApoE	5.44	0.02

^a^Likelihood ratio test for interaction. ^b^Adjusted for age and sex.

**Table 4:** Strata-specific hazard ratios based on ApoE genotype.

**APOE genotype**	**HR^c^**	**(95% CI)**
e2e2, e3e2, e3e3	1.15^***^	1.07–1.24
e4e3, e4e4	0.84	0.65–1.10

^*^p < 0.05; ^**^p < 0.01; ^***^p < 0.001. ^c^Adjusted for age and sex.

Subgroup analysis was done with stratified age samples. We found differential effect of SVI on mortality among different age groups. In middle aged group (50~64 years old), SVI was significantly associated with mortality (hazard ratio [HR]: 1.20, 95% CI: 1.01–1.42, *p* = 0.039) but not in older age group (≥65 years old). (Supplementary Table 2) Besides, we also tested the interaction of APOE and SVI in stratified age groups and found SVI interacts with APOE in older adults (Supplementary Table 3) while the interaction was not found in middle aged adults. Among older age group, the age-specific hazard ratio was calculated and SVI was significantly associated with mortality in non-ε4 allele group (hazard ratio [HR]: 1.12, 95% CI: 1.03–1.22, *p* = 0.006) (Supplementary Table 4).

## DISCUSSION

In this study, we constructed the SVI from a nationally representative population-based cohort in Taiwan, and the SVI developed from this study presented similar features to those noted in previous studies [12, 31]. This study clearly identified different demographic and clinical characteristics among participants with different SVI levels. SVI status significantly predicted all-cause mortality after adjustment for confounding factors, including ApoE and 5-HTTLPR genotypes. Nevertheless, some differences existed in the distribution of ApoE and 5-HTTLPR genotypes in association with SVI. Specifically, higher chances of harboring the ApoE ε4 allele were noted in the high SVI group, but no differences in 5-HTTLR were noted across different SVI levels. The mortality risk was significantly higher among ApoE ε4 noncarriers in the high SVI group than ApoE ε4 noncarriers in the lower SVI group and ApoE ε4 carriers with any SVI status. The results suggested the moderating effects of ApoE genotypes on SVI in predicting all-cause mortality in the study participants. However, a similar relationship was not observed between 5-HTTLPR genotypes and SVI.

In this study, the SVI was normally distributed, which is similar to that noted other previous studies [12]; however, the median SVI value (0.35) was higher than values reported from the Canadian Study of Health and Aging (0.25) and National Population Health Survey (0.28) [12]. A number of items selected to construct the SVI in this study were self-reported, which may be strongly influenced by the sociocultural context in Taiwan, and individuals tended to report lower satisfaction and social status [32]. On the other hand, older Taiwanese persons were less likely to receive higher education than older people in Western countries. The rapid social and economic transition of Taiwan in recent decades has also drastically changed the socioeconomic status of its current inhabitants [33]. The potential cohort effect may explain the higher SVI values noted in Taiwan compared with other Western countries. The dose-responsive effects between SVI and depressive symptoms, cognitive impairment, physical dependency, and multimorbidity were identified in this study, and these results were similar to those noted in previous studies [10, 34, 35].

Survival analysis and the Cox model found that the SVI significantly predicted all-cause mortality, as noted in previous studies [12, 36], and ApoE and 5-HTTLPR genotypes did not change the prediction of the SVI. Gene-environment interactions have gained extensive research attention in recent decades. Previous studies did not support associations between physical frailty and ApoE genotypes, but two recent studies confirmed the gene-environment interaction between ApoE genotypes and physical frailty [37]. Thibeau et al. reported the moderation effects of ApoE in the associations between physical frailty and memories in which frailty-accelerated memory loss is increased among ε4 carriers compared with non-ε4 carriers [19]. In contrast, with a larger study sample of older people with mild cognitive impairment, Ward et al. discovered a potentially protective role of the frailty index on dementia progression among ApoE ε4 carriers compared with non-ε4 carriers, which suggested interventions for frailty prevention may show greater benefit in ApoE non-ε4 carriers [38]. To the best of our knowledge, this is the first study using a SVI to explore gene-environment interactions to test the moderation effects of specific genotypes on all-cause mortality. We found a lower survival probability among participants with a high SVI who lacked the ApoE ε4 allele compared with the other groups (Figure 4B). Further interactions were confirmed based on the differential strata-specific hazard ratio. Among non-ε4 carriers, a higher SVI was associated with a higher all-cause mortality risk. Among ε4 carriers, the SVI lost its predictive ability, which suggested potential moderation effects of gene-environment interactions. Nevertheless, no such interaction was found among 5-HTTLPR genotypes.

In the subgroup analysis, we found that SVI predicted mortality differentially among different age groups. In the middle-age group, SVI significantly predicted mortality but the interaction with ApoE was not significant. On the other hand, in older group, SVI lost predictive ability on mortality risk but ApoE interacted with SVI. (Supplementary Tables 2 and 3) Therefore, we could extend our findings that SVI plays more important role on mortality risk among middle-aged people despite of ApoE genotype, and the moderation effect of ApoE on social vulnerability was greater in older age population. The potential explanation for the interaction between ApoE and social vulnerability may be that, among ApoE ε4 carriers, genetic matters more than social factors over the mortality risk; but among non-ε4 carriers, social factors plays greater roles in predicting over mortality, which indicate potential strategy for socially vulnerable older people.

The selection of ApoE and 5-HTTLPR genotypes to test the hypothesis of GxE interactions was based on the strong social impacts on mental health, including dementia and depression. The development of dementia and depression is multifactorial and certainly includes genetic and social factors. The dynamic interactions among genes, diseases, and social vulnerability over time contribute to the overall mortality risk in late life. Nevertheless, the results of this study again underscore the importance of social vulnerability in associations with all-cause mortality. The mortality risk of social vulnerability is moderated by ApoE but not 5-HTTLPR genotypes after adjustment for age and sex, which partly supported our research hypothesis regarding the GxE interaction. The results of this study implied the differential effects of specific genes on the GxE interaction in terms of the all-cause mortality risk. Although it has been reported that negative impacts of life stresses or trauma experiences on depression were moderated by 5-HTTLPR, the roles of overall social vulnerability may be more important in associations with mortality. Hence, continuing efforts to reduce social vulnerability may be the most critical determinant to promote healthy aging and healthy longevity.

Despite all research efforts made in this study, there were still some limitations. First, most variables used to construct the SVI were self-reported, and the background sociocultural context may bias the results. Second, outcome indicators, such as incident dementia, depression, incident disability, and healthcare utilization, were unavailable in this study, which limited the possibility of examining the roles of SVI and GxE interactions on these outcomes. Third, we were not able to explore the prognostic impacts of specific items not listed in the SEBAS questionnaire, e.g., health literacy, to construct a more comprehensive SVI. However, based on cumulative deficit theory, studies have indicated that the prediction model would be saturated if a specific number of selected variables were included, but a limited number of variables were included in our model. Therefore, the SVI constructed in this study should be sufficiently stable and accurate; however, some parameters were not included in the SEBAS questionnaire.

In conclusion, social vulnerability is a strong risk for all-cause mortality among community-dwelling middle-aged and older adults after adjustment for functional status and multimorbidity. Specific ApoE genotypes interact with social vulnerability in associations with mortality, whereas 5-HTTLPR genotypes do not. Strategies to promote healthy aging should be designed to reduce social vulnerability to maximize the intervention effects for healthy longevity.

## Supplementary Materials

Supplementary Tables
