# Supplementary material for: Differential moderation effects of ApoE and 5-HTTLPR genotypes on social vulnerability in predicting mortality among community-dwelling middle-aged and older adults: a nationwide population-based study
Source: Aging (Albany NY). 2021 Oct 14;13(19):23348–60. doi: 10.18632/aging.203629 (PMC8544323; doi:10.18632/aging.203629)
Supplement: Supplementary Tables [file aging-13-203629-s001.pdf]

SUPPLEMENTARY MATERIALS

Supplementary Tables

Supplementary Table 1. Items aggregated in the social vulnerability index from SEBAS 2006.

**Living situation**	
1.	Marital status
2.	Living alone
3.	Neighborhood
**Social support**	
4.	Felt lonely
5.	People weren’t nice to you
6.	Argument/disagreement with anyone in last 24 hr
7.	See your residence/community as safe
**Socially oriented activities of daily living**	
8.	Telephone use
9.	Get to places out of walking distance
**Leisure activities**	
10.	Play chess or cards with others
11.	Chat with relatives/friends, drink tea socially
12.	Jog, climb mountains, do outdoor activities with others
13.	Group activities: singing, dancing, tai chi
14.	Number of participating groups
**Empowerment, life control**	
15.	Do those close to you make too many demands on you?
16.	Felt joyful
17.	Felt life going well
18.	Agree that usually more good things than bad will happen
19.	Little control over things that happen to you
20.	What happens in future depends mostly on yourself
21.	Really no way you can solve some of your problems
22.	Little you can do to change important things in your life
23.	Can do just about anything i set my mind to
24.	Often feel helpless in dealing with problems of life
25.	Sometimes feel pushed around in life
26.	Anything else happen in last 24 hrs that was stressful
**Major life events and trauma events**	
27.	Major life events (number)
28.	Trauma events (number)
**Socio-economic status**	
29.	Are you satisfied with current living situation?
30.	Any difficulty meeting living expenses
31.	Self-reported socio-economic status
32.	Education level

Supplementary Table 2. Cox proportional hazard ratio of social vulnerability index (SVI) and all-cause mortality adjusted by age, sex and different genotypes, stratified by age ≧65 yrs and <65 yrs.

Variables	Age <65 yrs[a] (*N* = 501)		Age ≧65 yrs[a] (*N* = 484)	
	HR	95% CI	HR	95% CI
SVI	1.20	(1.01–1.42)*	1.07	(0.97–1.18)
Age	1.15	(0.98–1.34)	1.07	(1.02–1.13)**
Sex	0.35	(0.12–1.07)	0.51	(0.29–0.90)*
Education level	1.07	(0.36–3.24)	0.85	(0.47–1.53)
Multimorbidity	0.78	(0.28–2.17)	0.92	(0.49–1.73)
SPMSQ	1.92	(1.39–2.66)***	1.12	(0.93–1.35)
ADL	0.82	(0.54–1.24)	0.97	(0.81–1.19)
5-HTTLPR	1.00	(0.29–3.44)	1.32	(0.60–2.90)
APOE	1.04	(0.23–4.66)	0.59	(0.25–1.36)

*$p < 0.05$; **$p < 0.01$; ***$p < 0.001$. [a]Adjusted for age, sex, education level (>6 years), multimorbitidy (≥2 chronic conditions), SPMSQ, ADL, APOE and 5-HTTLPR genotypes.

Supplementary Table 3. Tests of interaction terms.

Social vulnerability index#ApoE[a]	LR test[b]	*p*-Value
<65 years old	0.29	0.59
≥65 years old	5.16	0.02

[a]Likelihood ratio test for interaction. [b]Adjusted for age and sex.

Supplementary Table 4. Strata-specific hazard ratios based on ApoE genotype in older adults (≥65 years old).

APOE genotype	HR[c]	(95% CI)
e2e2, e3e2, e3e3	1.12**	1.03–1.22
e4e3, e4e4	0.78	0.57–1.07

*$p < 0.05$; **$p < 0.01$; ***$p < 0.001$. [c]Adjusted for age and sex.
